# Integrated hybrid modelling of lignin bioconversion

**DOI:** 10.69997/sct.180358

**Published:** 2025-06-27

**Authors:** Sidharth Laxminarayan, Lily Cheung, Fani Boukouvala

**Affiliations:** a Georgia Institute of technology, School of Chemical and Biomolecular Engineering, Atlanta, GA, USA 30331

**Keywords:** Biosystems, Machine Learning, Dynamic Modelling, Lignin Valorization

## Abstract

Global biomanufacturing is projected to expand rapidly in the coming decade due to advancements in DNA sequencing and manipulation. However, the complexity of cellular behaviour introduces difficulty in modelling and optimizing biomanufacturing processes. Phenomenological models that represent the physics of the system in empirical equations suffer from poor robustness, while their machine learning (ML) counterparts suffer from poor extrapolative capability. On the other hand, hybrid models allow us to leverage both physical constraints and the flexibility of ML. This work describes a new approach for hybrid modeling that integrates the time-variant parameter estimation and ML model training into a singular step. We implement this approach on a proposed scheme for the cell-mediated conversion of a lignin derivative into a bioplastic precursor and show that our integrated hybrid model outperforms the traditional two-step hybrid, phenomenological and ML model counterparts. Lastly, we demonstrate how to execute an interpretability analysis on the ML component of the integrated hybrid model to reveal new physical insights that are then used to further improve model performance.

## Motivation & Background

**Figure 1.**
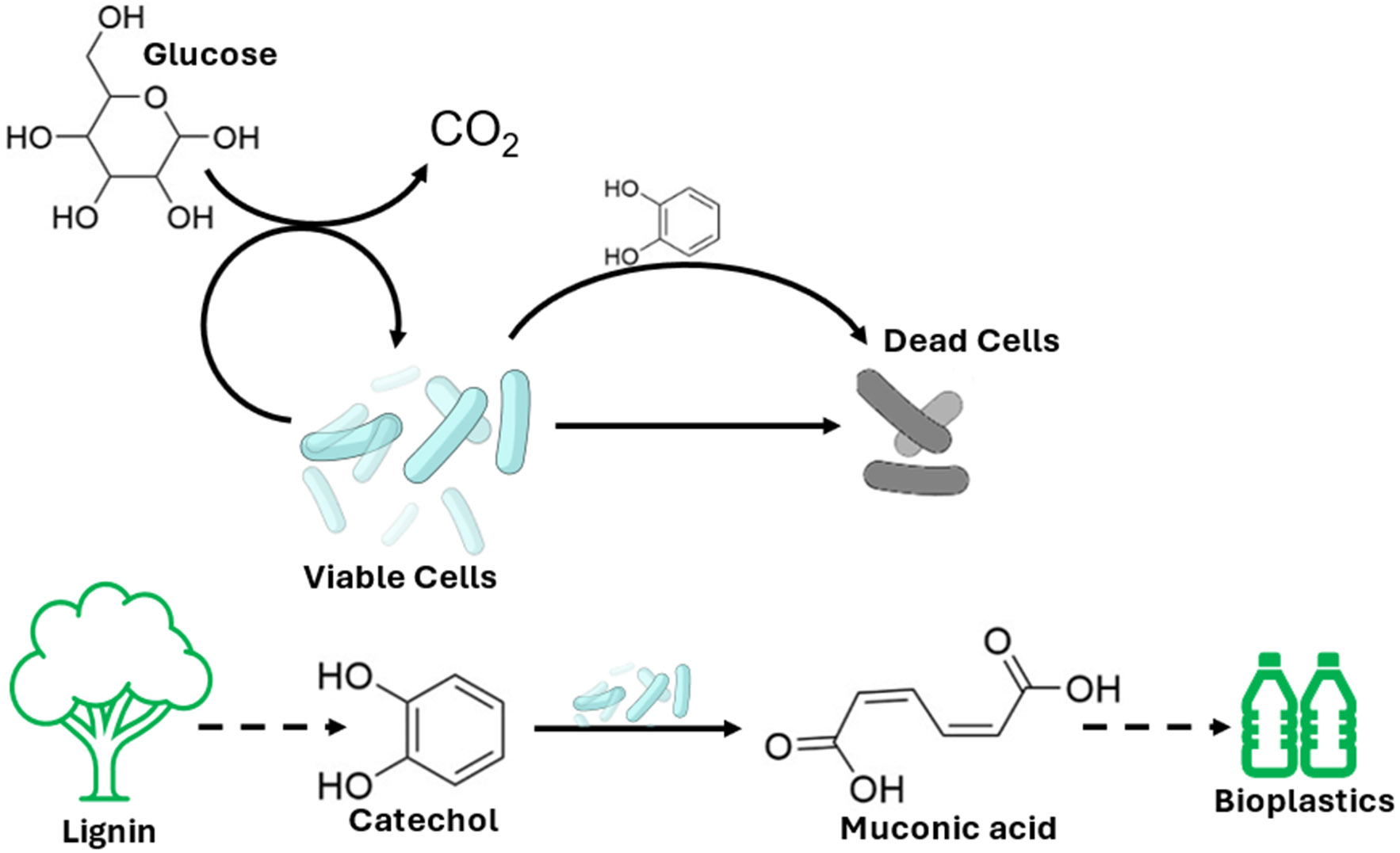
Conversion of lignocellulosic biomass into bioplastics by *P. putida*.

Growing need and sustainability concerns are increasing the strain on the traditional manufacturing pipelines for chemicals, drugs, and materials [1]. This has prompted increased interest on bio-centric manufacturing, as bioprocesses leverage the capability of microbial, plant, and animal cells to produce complex commercially relevant chemicals while enjoying a reduced carbon footprint, moderate reactor conditions, and high stereoselectivity [1]. Previously inaccessible renewable feedstocks can now be converted into bulk chemicals using engineered strains. Of particular interest is the soil bacterium *Pseudomonas putida*, which can valorize lignocellulosic biomass (Figure 1). Studies have demonstrated that *P. putida* can convert lignin into cis,cis-muconic acid (MA) [2]. MA can then be utilized to create polymers like Nylon and PET [2]. While the bacterial conversion of lignin into MA is documented, the kinetics is not well understood.

Cells are extremely complex systems with numerous reaction pathways, intermediates and products. Factoring in inter and intra-cellular relationships, it becomes intractable to simulate each cell and track metabolic dynamics [2]. This problem is countered using phenomenological models based on biological intuition that neglect informa- tion regarding biomass heterogeneity and metabolite influences on empirical parameters [3]. On the other hand, purely machine learning (ML) models have been shown to capture the nonlinear complexity of bioprocesses but have poor extrapolation and interpretability [3]. Additionally, experimental datasets are often sparse and noisy, resulting in poor model development and calibration, especially for ML models.

Hybrid models have been proposed to leverage the physical constraints of phenomenological models and the flexibility of ML ones [3]. This work explores an embedded hybrid model wherein ML models are utilized to capture the complexity of certain parameters “embedded” within the overarching ODE structure [4]. The hybrid modelling approach aids in capturing the complex relationships between external metabolites and bacteria physiology. Moreover, the embedded approach lends a better framework for interpretability.

To test our hybrid model, we outline a ground truth model that simulates the dynamics of the conversion of catechol, a lignin derivative, to MA by *P. putida*. The ground truth model also includes glucose as the representative carbon source for bacterial growth and the cell toxicity of catechol. A sparse and noisy dataset is then generated which mimics traditional experimental datasets.

This work will explore two scenarios for constructing hybrid models: A sequential method and an integrated method, which differ on how the parameters of the models are determined. Furthermore, we compare the extrapolative performance of the hybrid model to its phenomenological and ML counterparts. Finally, an interpretability study is performed on the ML components of the hybrid model to gain physical insight. Utilizing Captum python package, an Integrated Gradients (IG) analysis is performed which gives insight into how changes in the input affect changes in output for the ML component [5].

## Methods

### Ground truth model

A system of ODEs is proposed to simulate the dynamics of *P. putida* consuming glucose and converting catechol to MA in a batch process. The system is constructed based on biological principles and previous characterization of *P. putida* [2]. The following assumptions are made: (i) the growth rate of viable cells $\left(X_{v}\right)$ follows a logistic-Monod equation aimed at recapitulating the lag phase of batch cultures (Eq. 1 and 2); (ii) glucose $\left(C_{g}\right)$ promotes cell division while catechol $\left(C_{c}\right)$ inhibits it (Eq. 2); (iii) the cell death rate is composed of a zero-order, catechol-independent component and a catechol-dependent component represented with a Monod-style rate equation (Eq. 2); (iv) dead cells $\left(X_{d}\right)$ are not degraded and accumulate in the culture contributing to the total biomass $\left(X_{t}\right)$ (Eq. 4 and 5); (v) glucose is consumed for cell growth and to neutralize the toxic effect of catechol (Eq. 6); (vi) the generation of MA $\left(C_{m}\right)$ from catechol has non-growth associated and growth associated components (Eq. 7); and (vii) all the catechol consumed is converted to MA by the viable cells and no byproducts are formed (Eq. 8). Note that the following equation can be modified for batch and semi-batch by changing the spike glucose input $\left(C_{g}^{IN}\right)$, spike catechol input $\left(C_{c}^{IN}\right)$ and spike time-period (τ).


(1) $$\frac{dX_{v}}{dt}=\;\left(\mu_{gr}-\mu_{dt}\right)X_{v}$$

(2) $$\mu_{gr}=\;\left(1-\frac{X_{v}}{Y_{xg}C_{g}^{0}}\right)\left(\frac{\mu_{g}C_{g}}{C_{g}+K_{g}}\right)$$

(3) $$\mu_{dt}=\;k_{d}+\;\left(\frac{\mu_{c}C_{c}}{C_{c}+K_{c}}\right)$$

(4) $$\frac{dX_{d}}{dt}=\mu_{dt}X_{v}$$

(5) $$X_{t}=\;X_{v}+X_{d}$$

(6) $$\frac{dC_{g}}{dt}=\;-\left(\frac{\mu_{gr}}{Y_{xg}}+\frac{m_{k}C_{c}}{C_{c}+k_{cm}}\right)X_{v}+C_{g}^{IN}f(t)$$

(7) $$\frac{dC_{m}}{dt}=Y_{mx}\left(\mu_{gr}+\beta \right)X_{v}\frac{C_{c}}{C_{c}+k\_cr}\;$$

(8) $$\frac{dC_{c}}{dt}=-\frac{dC_{m}}{dt}+C_{c}^{IN}f\left(t\right)$$

(9) $$f\left(t\right)=\;\sum_{k=0}^{Z}\delta (t-\tau k)$$

**Figure 2.**
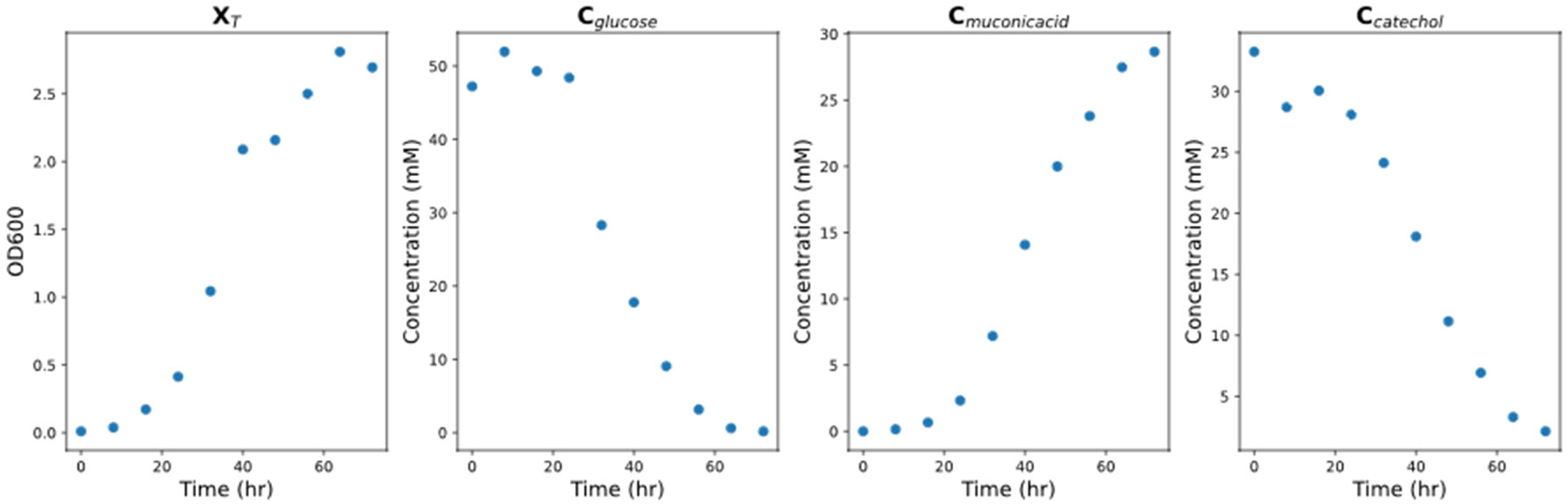
Ground truth model of the batch MA production bioprocess.

Training data is generated by assuming batch operation. A 72-hour batch process is simulated with a 10-point discretization. To better reflect limitations in data acquisition commonly experienced in biomanufacturing, only data on the total biomass, glucose, catechol and MA concentrations are used for training (Fig. 2). For instance, only total biomass is easily measured during culturing (e.g., by measuring changes on optical density) while distinguishing viable and dead cells is more challenging. Glucose, catechol and MA are generally quantified with either chromatographic or biochemistry analyzers, which are expensive and time consuming to operate thereby limiting the amount of data collected.

### Phenomenological model construction

This model reflects the traditional reactor kinetics style system of ODEs that are generally constructed to capture bioprocess dynamics. The data acquisition limitations highlighted in the previous section along with relevant literature informed the model construction. Combining dynamic observations of (Fig. 2) and literature studies [2], the following ODEs for the phenomenological model are created: (i) The total biomass growth is tied positively to glucose and inhibited by catechol via Monod kinetics (Eq.11); (ii) The MA production is observed to occur in both the growth and stationary phases (Eq. 13); (iii) The MA concentration increased at the same rate as the catechol one decreased (Eq. 14)

(10) $$\frac{dX_{t}}{dt}={\mu_{gr}X}_{t}$$

(11) $$\mu_{PM}=\;\left(\frac{\mu_{g}C_{g}}{C_{g}+K_{g}}\right)-\;\left(\frac{\mu_{c}C_{c}}{C_{c}+K_{c}}\right)$$

(12) $$\frac{dC_{g}}{dt}=\;-\left(\frac{\mu_{gr}}{Y_{xg}}\right)X_{t}\;+C_{g}^{IN}f(t)$$

(13) $$\frac{dC_{m}}{dt}=Y_{mx}\left(\mu_{gr}+\beta \right)X_{t}\frac{C_{c}}{C_{c}+k\_cr}\;$$

(14) $$\frac{dC_{c}}{dt}=-\frac{dC_{m}}{dt}+C_{c}^{IN}f\left(t\right)$$

(15) $$f\left(t\right)=\;\sum_{k=0}^{Z}\delta (t-\tau k)$$

The spike input of glucose and catechol are set to zero for the training data. Note how the growth of the biomass in the phenomenological model closely mirrors the ground truth model. The parameters of the phenomenological model are estimated using a Pyomo DAE formulation, which utilized an IPOPT multistart solver with fifty initializations [5].

### Hybrid model structure – Sequential vs Integrated

The construction of the hybrid model entrails the following steps: (a) time-variant parameter estimations (TVPE), (b) ML model parameter regression and (c) ODE-ML model integration. The integrated method differs from the sequential one as it performs steps (a) and (b) together. The equations shown here reflect the hybrid construction methodology. The observations outlined in the previous section are used to inform the overarching ODE structure. An assumption is made that the growth rate did not have a straightforward relationship between glucose and catechol. An artificial neural network (ANN) is used to model the growth rate which used glucose, catechol, MA and total biomass as inputs. Equations (16-19) highlight the hybrid model structure.


(16) $$\frac{dX_{t}}{dt}={\mu_{ML}X}_{t}$$

(17) $$\mu_{ML}=\;ANN(X_{t},C_{g},C_{c},\;C_{m})$$

(18) $$\frac{dC_{g}}{dt}=\;-\left(\frac{\mu_{ML}}{Y_{xg}}\right)X_{t}+C_{g}^{IN}f\left(t\right)$$

(19) $$\frac{dC_{m}}{dt}=Y_{mx}\left(\mu_{ML}+\beta \right)X_{t}\frac{C_{c}}{C_{c}+k\_cr}\;$$

Equations 14, 15 are included in the hybrid model to track the dynamics of catechol and the spike input frequency. The sequential hybrid modelling structure initially calculates the TVPE and then trains a ML model outside. Notice that within the initial optimization framework the TVPE is calculated as a discrete value at different time steps. A multi-objective optimization problem is formulated with a state space MSE and a smoothing index (sum of second derivatives) for the time variant parameters. The formulation shown below shows the sequential hybrid model’s TVPE.


(20) $$\min_{\theta^{TV},\theta_{TIV}}\;w_{M}MSE+w_{s}SM$$

(21) $$s.t.\frac{dC_{si}}{dt}=f_{ODE}(C_{xi},\theta_{TIV},\theta_{i}^{TV}),\forall \;x=[0,S]\forall \;i=[0,N]$$

(22) $$MSE=\frac{1}{NtS}\sum_{i=1}^{N}\sum_{j=0}^{t}\sum_{s=0}^{S}{\left(C_{sji}^{pred}-C_{sji}^{act}\right)}^{2}$$

(23) $$SM=\frac{1}{Nt}\sum_{i}^{N}\sum_{j}^{t-2}{\left(\theta_{ij+1}^{TV}-2\theta_{ij}^{TV}+\theta_{ij-1}^{TV}\right)}^{2}$$

(24) $$\theta_{it}^{TV},\theta_{TIV}\geq 0$$

N represents the number of experimental datasets, t is the number of time points in each dataset and S is the number of dynamic species. $f_{ODE}$ represent the hybrid system of ODE captured by Eqs (14-19). $\theta_{TIV}$ are the time invariant parameters in Eqs (14-19) while $\theta^{TV}$ represents time variant parameters. The integrated hybrid modelling structure simultaneously estimates the growth parameter while also training the ANN model to capture the parameter behavior. The optimization formulation shown below details how the ML model is trained and the TVPE is performed.


(25) $$\underset{W,b,\theta_{\mathrm{TIV}}}{\min}\;\;\frac{1}{NtS}\sum_{i=1}^{N}\sum_{j=0}^{t}\sum_{s=0}^{S}{\left(C_{sji}^{pred}-\;C_{sji}^{act}\right)}^{2}$$

(26) $$s.t.\;\frac{dC_{si}}{dt}=f_{ODE}\left(C_{xi},\theta_{TIV},\;\theta_{i}^{TV}\right),\forall \;x=\left[0,\;S\right]\forall \;i=[0,\;N]$$

(27) $$\theta_{i}^{TV}=f_{ML}\left(W,b,C_{xi}\right)\;\forall \;i=0\;to\;N$$

(28) $$\theta_{TIV}\geq 0,\;\;W,\;b\;\subset [-1,\;1]$$

$f_{ML}$ represents the ANN whose weights and biases (W & b) are used to model the physics of the $\theta^{TV}$. The sequential hybrid modelling structure ignores the second equality constrain shown in Eq. (27) which captures the relationship between the time variant parameter and the ML model. Note that both the integrated and sequential method has hyperparameters which need to be tuned. For both the integrated and sequential scenarios, the IPOPT multistart solver with fifty different initializations is used to solve the Pyomo DAE formulation [5].

### Black box model construction

The black box model is constructed using an ANN surrogate. The model inputs are the initial condition and time. A preprocessing step is performed where the inputs and outputs are scaled using a Z-score normalization. A rigours 5-fold cross validation and grid search is performed for tuning the hyperparameters of the system. The activation function, the number of hidden layers, the number of nodes, and the strength of the L2 regularization are all varied.


(29) $$\left(X_{t}^{t},C_{g}^{t},C_{m}^{t},C_{c}^{t}\right)=\;ANN\left(X_{t}^{0},C_{g}^{0},C_{m}^{0},C_{c}^{0},t\right)$$

### Extrapolative performance comparison

The initial data is generated by performing a Latin hypercube sampling of three initial conditions in the following ranges of initial glucose and catechol concentration: [50 mM, 70 mM] and [25 mM, 35 mM] with a constant total biomass concentration of 0.01 OD600. The performance of all models is evaluated utilizing an initial condition extrapolation for a fed-batch bioprocess. Note how in the training data the spike inputs are set to zero, however for the performance testing the spike inputs are respectively set to 10 mM and 3 mM for glucose and catechol with a time-period of 25 hrs. In the extrapolative scenarios, the glucose and catechol initial concentrations are varied from [20 mM, 100 mM] and [5 mM, 55 mM], respectively. Heatmaps and distribution curves of $R^{2}$ values are generated to compare the performance of different models and highlight performance in edge-cases.

### Interpretability analysis

Utilizing the Captum software, the ANN growth rate in Eq. 17 is analyzed and some of the model behaviours are explained [6]. A primary attribution analysis called Integrated gradients (IG) analysis is performed which tells the contribution of each input to the output of the model. The higher the attribution scores the more the input contributes to the model output [6]. Positive attribution means the output increase with the increase of the input and vice versa [6]. The attribution is calculated utilizing an integrated gradients technique.

### Improved hybrid model

Based on some insight from the ML interpretability analysis, new physical insights are integrated into the hybrid model to improve its overall performance. The physical insight added to the hybrid model is the Monod-kinetics relationship between biomass growth and glucose. The following ODE equations shown details the improved hybrid model. The improved hybrid model utilizes an ANN to model the fraction of viable biomass in the reactor. Equations 14, 15 are included in the improved hybrid model to track the dynamics of catechol and the spike input frequency.


(30) $$\frac{dX_{t}}{dt}={{\mu f}_{v}X}_{t}$$

(31) $$f_{v}=\;ANN(X_{t},C_{g},C_{c},\;C_{m})$$

(32) $$\mu =\;\left(\frac{\mu_{g}C_{g}}{C_{g}+k_{g}}\right)$$

(33) $$\frac{dC_{g}}{dt}=\;-\left(\frac{\mu }{Y_{xg}}\right){f_{v}X}_{t}+C_{g}^{IN}f\left(t\right)$$

(34) $$\frac{dC_{m}}{dt}=Y_{mx}\left(\mu +\beta \right){f_{v}X}_{t}\frac{C_{c}}{C_{c}+k\_cr}\;$$

## Results and Discussion

### Sequential vs Integrated hybrid model construction

The hyperparameter tuning for the integrated model is a 3-fold cross validation to determine the ANN architecture. The hyperparameter tuning for the sequential model consist of two-steps: (i) a 3-fold cross validation to determine the smoothing weight $\left(w_{s}\right)$ and (ii) a 5-fold cross validation to determine the ANN architecture. To simplify the hyperparameter search, the MSE weight $\left(w_{M}\right)$ is set to a constant of unity. The performance of the sequential model is highest at a smoothing weight of 10. Following this, a grid search is done to determine the ANN architecture by varying the activation function, number of layers and nodes and L2 regularization weight.

**Figure 3.**
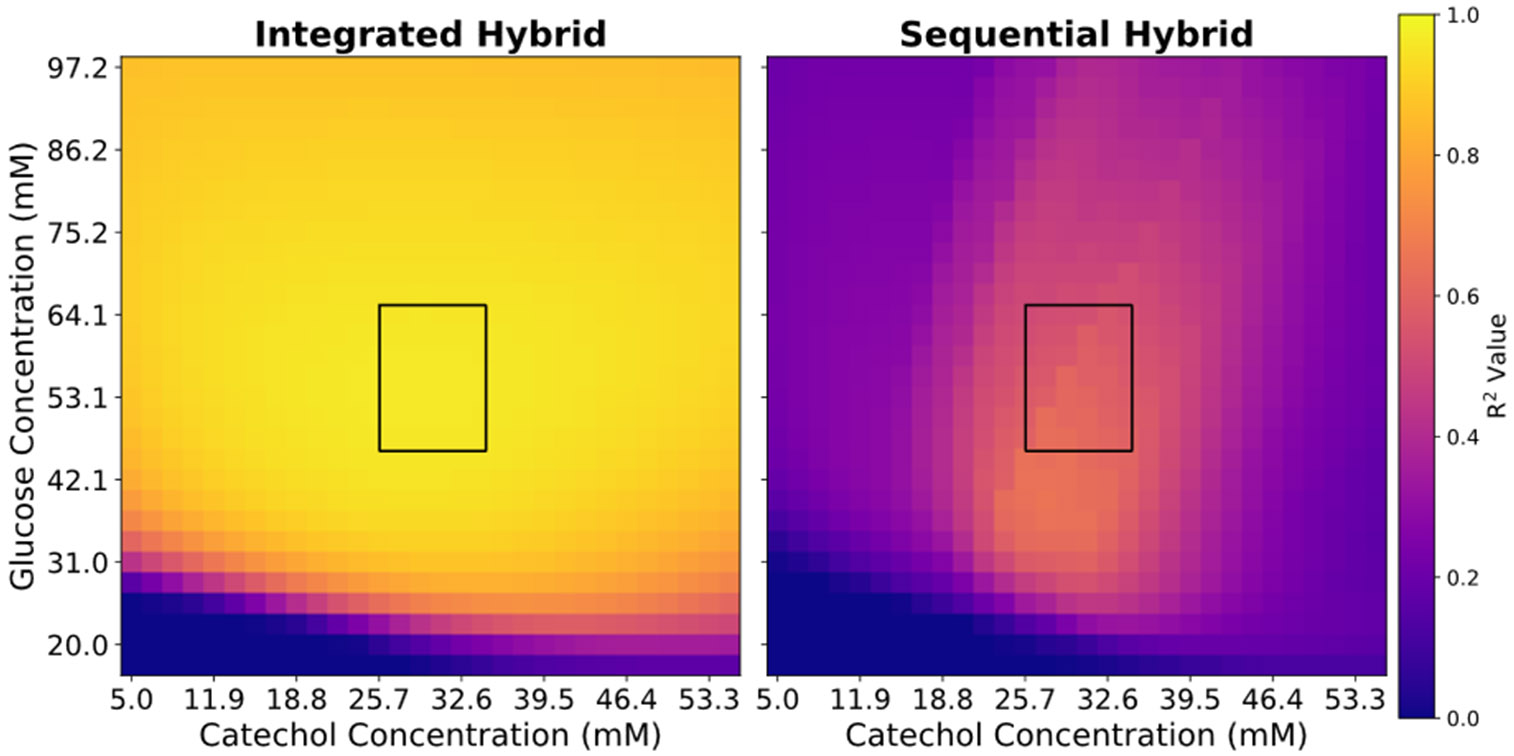
Extrapolative performance heatmap comparing the integrated and squential models.

The integrated model outperforms the sequential one across all points of extrapolation (Figure 3). The heatmap can be divided into three broad regions: (I) high glucose, (II) low-glucose and low-catechol, and (III) low-glucose and high-catechol. The integrated models performs better than the sequential model in region (I) and (III) but suffers in region (II). In fact, the integrated model performs its best predictive performance in region (I) given the strong effect of high glucose concentrations on the growth rate. However, in region (II) the low glucose compounded with low catechol concentrations decreases the fraction of viable biomass which decreases growth rate. The integrated model overestimates the growth rate in this region. These results agree with our ground truth model which has a stunted growth rate at low glucose and low catechol concentrations, thereby negatively affecting the biomass accumulation.

### Hybrid model extrapolative performance

The previous section shows the performance of the integrated hybrid model to be better than the sequential hybrid one. Thus, the following sections will exclusively utilize the integrated hybrid model for comparison with the phenomenological and black box models.

**Figure 4.**
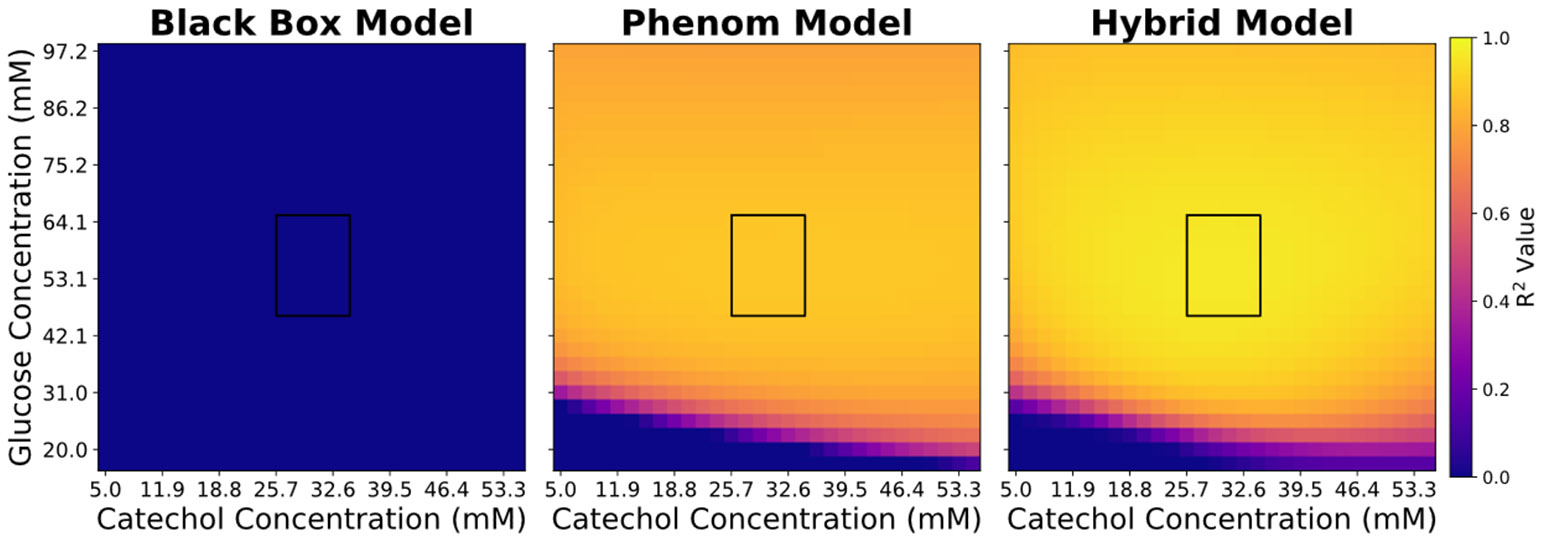
Extrapolative performance heatmap comparing the ML, phenomenological and hybrid models

On average, the hybrid model outperforms both the phenomenological and back box models in extrapolation (Figure 4). The black box model had extremely poor extrapolative performance; while it captured the intricacies of the training data, it struggled to extrapolate the behaviour. The hybrid model outperforms the phenomenological model in regions (I & III). However, it is extrapolative performance slightly suffers in regions (II) compared to its phenomenological counterpart, given that the latter has the physical insight which allows it to better anticipate the contribution of catechol in low glucose environments.

**Figure 5.**
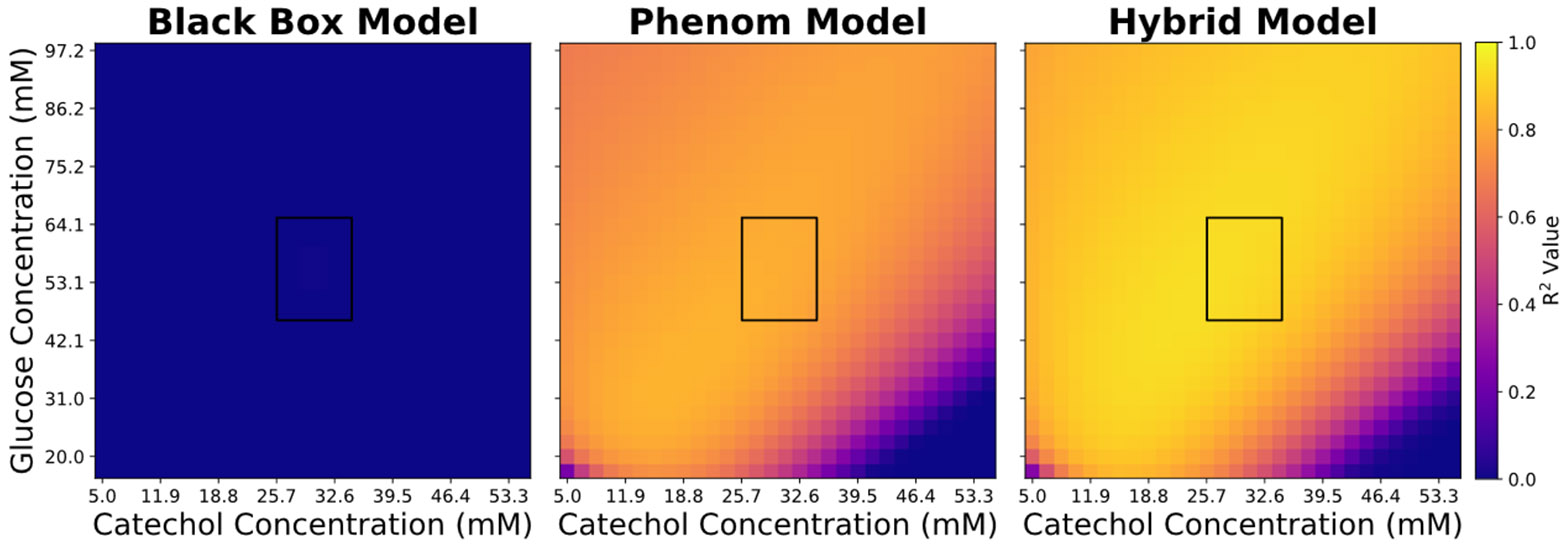
Extrapolative performance boxplot comparing the ML, phenomenological and hybrid models for greater contribution of maintenance coefficient effect on glucose

Next, we sought to test versatility of the hybrid model. The glucose consumption in the ground truth model (Eq. 6) has two contributing terms: biomass growth and maintenance. The maintenance is tied to concentration of the catechol concentration. The hybrid model and the phenomenological model are formulated with the assumption that the maintenance term is not a huge contributor to glucose consumption. The versatility of the hybrid modelling structure is tested by increasing the contribution of the glucose consumption to match the growth-related glucose consumption. Despite the change in the physics of the ground truth model, Eq. 10-19 are not changed. The hybrid model still outperforms both the phenomenological and black box model in all the regions (Figure 5), showing that the versatility of the embedded ANN can capture the intricacies of the growth rate while also accounting for the additional strain of maintenance. The hybrid model’s performance suffers in region (III); however, a trend observed to occur in the phenomenological model as well. The higher impact of catechol reduces the fraction of viable cells thereby overestimating the metabolic activity of the biomass to convert catechol into MA. This analysis demonstrates the flexibility of the integrated hybrid modelling structure to adapt to changes in the underlying physics of the system.

### Hybrid model interpretability

**Figure 6.**
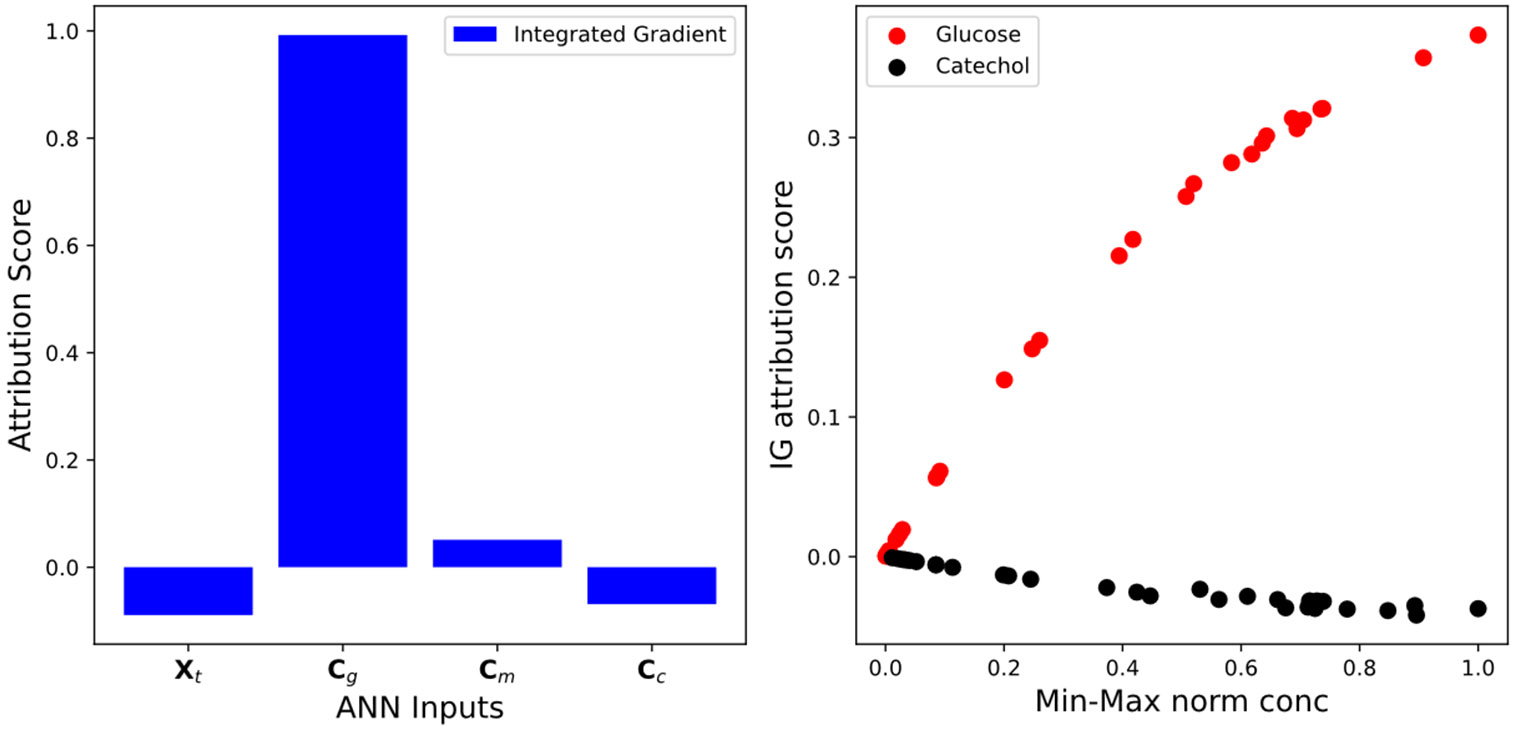
Primary attribution scores for the ML model in the hybrid ODE for training data. Model attribution score for changes in glucose and catechol concentration

The hybrid model still has an embedded ANN piece within its structure and understanding the physics that is captured by the ANN component could aid in model refinement and experimental design. The bar graph shows the attribution score for the ANN component of the hybrid model. Note, the primary contributor to the model’s predictive performance is the glucose and catechol concentrations. The glucose has a positive effect while catechol has a negative effect. This aligns with the physics that is present in the ground truth model. When we further analyze the attribution score of glucose as the concentration changes, further biophysical insights are caught. Fig. 6 looks at the glucose attribution score as the normalized glucose and catechol concentration changes. An important trend is how the attribution versus glucose plot mirrors the relationship between growth rate and substrate for Monod-style growth kinetics. Similarly note that catechol attribution score increases in magnitude as the concentration increases.

The attribution score analysis presented above can function as first step in constructing more accurate empirical models to capture unexplained phenomenological behaviour. With the aid of the ML model interpretability, we could implement a build-train-improve cycle to incorporate and improve the physics captured by the hybrid model. Using the information presented from the Captum analysis, the new observed physical trends could be incorporated into its physical constraints.

### Improved hybrid models with increased physics embedding

Based on the interpretability analysis, a Monod style relationship between glucose and growth rate is determined and incorporated into the improved hybrid model (Eq. 28-32). The improved hybrid model utilizes the ANN flexibility to capture the fraction of viable biomass in the system, which is the only entity metabolizing both glucose and catechol. Consequently, the aim of the improved hybrid is capturing this fact even though only total biomass (viable plus dead cells) is used for training. As seen on the performance distributions plots below, the improved hybrid models outperformed the original hybrid model due to an increased accuracy in low-glucose high-catechol environments. The improved model performs better in regions where the original hybrid one struggles. Note the thinner and taller distribution peak of the improved model in high R^2^ value range. Since some burden is alleviated from the ANN component by incorporating learned physics insight, the black-box part of the improved hybrid model has less impact on the generalizability of the overall prediction. This two-step approach to hybrid modeling highlights how the black-box components, coupled by quantitative interpretability analysis could expedite model development.

## Conclusion

The above results outline the implementation of a hybrid modelling strategy to capture the dynamics of a bioprocess. A case study of *P. putida* consuming glucose and converting catechol to MA, a bioplastics precursor, is studied. The biomass growth in the hybrid model is captured utilizing an ANN. A new hybrid model construction strategy is introduced which integrated the TVPE and the ML model training to occur in one step. Various models are evaluated on a fed-batch extrapolative reactor at various initial glucose and catechol concentrations. The versatility of the hybrid model is also assessed by increasing the influence of various physical phenomena in the ground truth model. The integrated hybrid model outperforms its sequential counterpart as well as commonly used phenomenological and ML models. An attribution analysis is performed to lend interpretability to the ANN piece of the hybrid model, revealing buried ground truth behaviour, which is then used to guide a model improvement cycle. The ultimate improved hybrid model is then shown to reduce the computational load of the ANN by incorporating previously discovered biophysical insights and to extrapolation performance.

## Supplementary Material

Code

The GitHub page has the code, data and figures shown in this paper: Escape35_SL_code.

## Figures and Tables

**Figure 7.**
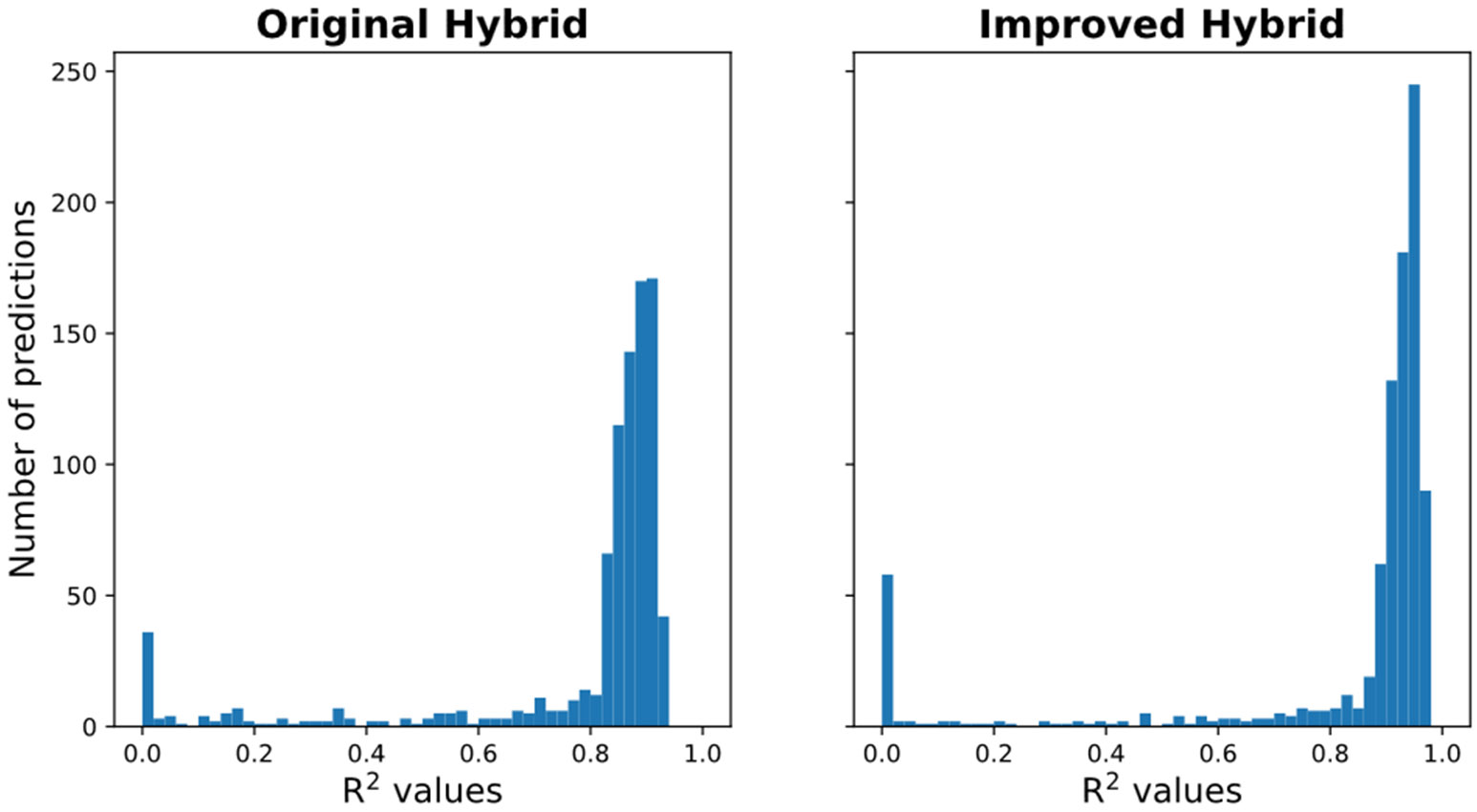
Extrapolative performance distributions for original hybrid and improved hybrid models.
